# A Test for Discriminating Between Members of the Odd Weibull-G Family of Distributions

**DOI:** 10.1155/2024/9423417

**Published:** 2024-11-05

**Authors:** Boikanyo Makubate

**Affiliations:** Department of Mathematics & Statistical Sciences, Botswana International University of Science and Technology, P. Bag 16, Palapye, Botswana

**Keywords:** discrimination between distributions, maximum entropy principle, odd Weibull-G family of distributions, Shannon entropy, 62E10, 62F30

## Abstract

The Odd Weibull-G (OWG) family of distributions has been discussed earlier in the literature. This family of distributions provides a “better fit” in certain practical situations. In a similar fashion, the OWG family of distributions is defined in this article. A method of moments estimator based on the maximum entropy principle is proposed for the discrimination of two members of the OWG family of distributions.

## 1. Introduction

There have recently been attempts to create new families of probability distributions to represent and understand real-world phenomena [1–7]. Generating new distributions allows researchers and practitioners to develop more accurate models that can capture the underlying characteristics of different datasets. One such illustration is a large family of univariate distributions derived from the Weibull distribution that Bourguignon, Silva, and Cordeiro [8] proposed. Bourguignon, Silva, and Cordeiro [8] extend any continuous baseline distribution by two additional parameters using the T-X technique [9]. The two additional parameters control the skewness and the kurtosis of the distribution. For any baseline cumulative distribution function (cdf), *G*(*x*; *ζ*), which depends on a parameter vector *ζ*, following the notation of Bourguignon, Silva, and Cordeiro [8], the cdf and probability density function (pdf) of the Odd Weibull-G (OWG) family of distributions is defined by

(1) $$\begin{array}{c} F\left(x;\beta_{1},\beta_{2},\zeta \right)=1-\exp\left\{-\beta_{1}{\left[\frac{G\left(x;\zeta \right)}{\bar{G}\left(x;\zeta \right)}\right]}^{\beta_{2}}\right\} \end{array}$$

and

(2) $$\begin{array}{c} f\left(x;\beta_{1},\beta_{2},\zeta \right)=\beta_{1}\beta_{2}g\left(x;\zeta \right)\frac{G{\left(x;\zeta \right)}^{\beta_{2}-1}}{\bar{G}{\left(x;\zeta \right)}^{\beta_{2}+1}}\exp\left\{-\beta_{1}{\left[\frac{G\left(x;\zeta \right)}{\bar{G}\left(x;\zeta \right)}\right]}^{\beta_{2}}\right\}, \end{array}$$

respectively, where $\overset{¯}{G}\left(x;\zeta \right)=1-G\left(x;\zeta \right)$, *g*(*x*; *ζ*) = *dG*(*x*; *ζ*)/*dx*, *x* > 0, and *β*_1_ and *β*_2_ are positive parameters. Model (2) has the advantage that a vast family of distributions can be formed from any continuous distribution, *G*(*x*; *ζ*), with the parameters *β*_1_ and *β*_2_ regulating the skewness and the kurtosis. In their article, Bourguignon, Silva, and Cordeiro [8] showed that this family of distributions provides a better fit than other commonly used distributions. In the literature, families of Weibull related distributions have also been addressed, for example, the new Weibull generalized-G by Oluyede, Sengweni, and Makubate [10]; the Weibull normal distribution by Famoye, Akarawak, and Ekum [11]; the Weibull exponential distribution by Oguntunde et al. [12] and the Weibull Dagum by Tahir et al. [13]; to mention a few. The class of distributions for the special case of *β*_2_ = 1 is referred to as the Odd Exponential-G (OEG) family of distributions with cdf and pdf given by

(3) $$\begin{array}{c} F_{OWG}\left(x;\beta_{1},\zeta \right)=1-\exp\left\{-\beta_{1}\left[\frac{G\left(x;\zeta \right)}{\bar{G}\left(x;\zeta \right)}\right]\right\} \end{array}$$

and

(4) $$\begin{array}{c} f_{OWG}\left(x;\beta_{1},\zeta \right)=\beta_{1}g\left(x;\zeta \right)\frac{1}{\bar{G}{\left(x;\zeta \right)}^{2}}\exp\left\{-\beta_{1}\left[\frac{G\left(x;\zeta \right)}{\bar{G}\left(x;\zeta \right)}\right]\right\}, \end{array}$$

respectively.

Determining whether specific data can be presumed to have come from one of the two provided arbitrary probability distributions has been a long-standing problem in statistics. Atkinson [14, 15], Chambers and Cox [16], Chen [17], Cox [18, 19], Dumonceaux and Antle [20], Dyer [21], Gupta and Kundu [22, 23], Jackson [24], Kundu et al. [25], Lee and Max [26], and Raqab, Al-Awadhi, and Kundu [27] all had earlier discussed and provided respective solutions for this problem. In particular, Dumonceaux and Antle [20], Lee and Max [26], and Gupta and Kundu [22] derived methods of choosing between the Weibull and the log-normal distributions, the Weibull and the gamma distributions, and the Weibull and the generalized exponential distributions, respectively.

Raqab, Al-Awadhi, and Kundu [27] considered discriminating among three positively skewed models being Weibull, log-normal and log-logistic distributions. In this article, a method of moments estimator based on the maximum entropy principle [28] is proposed for the discrimination of two members of the OWG family of distributions. A not very dissimilar idea was earlier proposed by Zografos and Balakrishnan [29] in discriminating between beta generated models and gamma generated models, respectively.

Due to the increasing applications of the OWG family of distributions [11–13], in this article, special attention is given to developing a test that would allow mathematicians to determine whether a sample taken at random from (4) is coming from a specific *G*(*x*; *ζ*) distribution. In this research, it is observed that the difference of the Shannon entropy [30] of any two given members of the OWG can be used to discriminate between the members of the family in (4). In addition, it is possible to construct an analytical form for the Shannon entropy of some members of the OWG family. Earlier, Huang et al. [31], although on a different problem, discussed in their article some entropy based methods.

The paper is outlined as follows. The Shannon entropy of the OWG, with pdf in (4) is presented in Section 2. The Shannon entropies of three univariate distributions produced by model (4) will be determined in a closed form and provided as examples. In Section 3, a procedure for discriminating between members of the OWG family is proposed, followed by conclusions.

## 2. Shannon Entropy

Shannon entropy, named after the mathematician and information theorist Claude Shannon [30], is a measure of information content in a given set of data. It provides a quantitative measure of the average amount of information needed to specify an outcome from a set of possibilities. Shannon entropy has applications in various fields, including information theory, data compression, cryptography, and machine learning [29]. The Shannon entropy of a continuous distribution with density, let us say *f*(*x*), is defined by

(5) $$\begin{array}{c} S\left(f\right)=-\int_{-\infty }^{\infty }f\left(x\right)\ln\;f\left(x\right)dx. \end{array}$$

Thus, the Shannon entropy of the OWG family of distributions, with pdf in (4), is given by

(6) $$\begin{array}{c} S\left(f_{OWG}\right)=-\ln\;\beta_{1}-E_{OWG}\left[\ln\;g\left(x;\zeta \right)\right]+2E_{OWG}\left[\ln\bar{G}\left(x;\zeta \right)\right]+\beta_{1}E_{OWG}\left[\frac{G\left(x;\zeta \right)}{\bar{G}\left(x;\zeta \right)}\right], \end{array}$$

where *E*_*OWG*_ denotes expectation under the pdf in (4).

**Lemma 1 .**

Let the random variable *X* be described by the OWG family in (4). Then, the random variable $0<Z=G\left(x;\zeta \right)/\overset{¯}{G}\left(x;\zeta \right)<\infty$ has an exponential distribution with pdf

(7) $$\begin{array}{c} f\left(z;\beta_{1}\right)=\beta_{1}\;\exp\left(-\beta_{1}z\right). \end{array}$$

**Proof**

We let *Z* = *G*(*x*; *ζ*)/1 − *G*(*x*; *ζ*). Thus, the Jacobian is given by *dx*/*dz* = (1 − *G*(*x*; *ζ*))^2^/*g*(*x*; *ζ*). Substituting *Z* into (4) and multiplying by the Jacobian yields the pdf of the random variable *Z* as

(8) $$\begin{array}{c} f\left(z;\beta_{1}\right)=\beta_{1}\;\exp\left(-\beta_{1}z\right). \end{array}$$

**Lemma 2 .**

If *G*(*x*; *ζ*) and *g*(*x*; *ζ*) are any arbitrary continuous cdf and pdf, respectively, then

a. $E_{OWG}\left[G\left(x;\zeta \right)/\overset{¯}{G}\left(x;\zeta \right)\right]=1/\beta_{1}$;
b. $E_{OWG}\left[\ln G\left(x;\zeta \right)/\overset{¯}{G}\left(x;\zeta \right)\right]=-\gamma -\ln\;\beta_{1}$;
c. $E_{OWG}\left[\ln\overset{¯}{G}\left(x;\zeta \right)\right]=-e^{\beta_{1}}Ei\left(1,\beta_{1}\right)$;
d. *E*_*OWG*_[ln *g*(*x*; *ζ*)] = *E*_*Z*_[ln *g*(*G*^−1^[*z*/(1 + *z*)])],

where *Z* has an exponential distribution with the rate parameter *β*_1_, *E*_*OWG*_ denotes expectation under the pdf in (4), *γ* ≈ 0.5772 is the Euler–Mascheoni constant, and

(9) $$\begin{array}{c} Ei\left(1,\beta_{1}\right)=\int_{\beta_{1}}^{\infty }\;\ln\left(\frac{z}{\beta_{1}}\right)e^{-z}dz<\infty . \end{array}$$

**Proof**

To verify Parts (a), (b), (c), and (d), we let *Z* = *G*(*x*; *ζ*)/1 − *G*(*x*; *ζ*) to obtain

(10) $$\begin{array}{c} E_{OWG}\left[\frac{G\left(x;\zeta \right)}{\bar{G}\left(x;\zeta \right)}\right]=E_{Z}\left[z\right]=\frac{1}{\beta_{1}}, \end{array}$$

(11) $$\begin{array}{c} E_{OWG}\left[\ln\frac{G\left(x;\zeta \right)}{\bar{G}\left(x;\zeta \right)}\right]=\beta_{1}\int_{0}^{\infty }\;\ln\left(z\right)e^{-\beta_{1}z}dz=-\gamma -\ln\;\beta_{1}, \end{array}$$

(12) $$\begin{array}{c} E_{OWG}\left[\ln\bar{G}\left(x;\zeta \right)\right]=\beta_{1}\int_{0}^{\infty }\;\ln\left(\frac{1}{1+z}\right)e^{-\beta_{1}z}dz=-e^{\beta_{1}}Ei\left(1,\beta_{1}\right), \end{array}$$

(13) $$\begin{array}{c} {E_{OWG}\left[\ln\;g\left(x;\zeta \right)\right]=\int }_{0}^{\infty }\;\ln\;g\left(G^{-1}\left[\frac{z}{\left(1+z\right)}\right]\right)\beta_{1}\;\exp\left(-\beta_{1}\right)dz \end{array}$$

**Lemma 3 .**

The Shannon entropy of the OWG distribution with pdf in (4) is given by

(14) $$\begin{array}{c} S\left(f_{OWG}\right)=1-\ln\;\beta_{1}-2e^{\beta_{1}}Ei\left(1,\beta_{1}\right)-E_{Z}\left[\ln\;g\left(G^{-1}\left[\frac{z}{\left(1+z\right)}\right]\right)\right]. \end{array}$$

where Z has an exponential distribution with the rate parameter *β*_1_, and

(15) $$\begin{array}{c} Ei\left(1,\beta \right)=\int_{\beta_{1}}^{\infty }\;\ln\left(z/\beta_{1}\right)e^{-z}dz. \end{array}$$

**Proof**

It is readily obtained by applying Lemma 2 into equation (6).

**Lemma 4 .**

The pdf of OWG defined in (4) is the unique solution of the optimization problem

(16) $$\begin{array}{c} f_{OWG}\left(x\right)=\arg\max_{f}S\left(f\right), \end{array}$$

under the constraints

1. $E_{f}\left[G\left(x;\zeta \right)/\overset{¯}{G}\left(x;\zeta \right)\right]=1/\beta_{1}$;
2. $E_{f}\left[\ln\overset{¯}{G}\left(x;\zeta \right)\right]=-e^{\beta_{1}}Ei\left(1,\beta_{1}\right)$;
3. *E*_*f*_[ln *g*(*x*; *ζ*)] = *E*_*Z*_[ln *g*(*G*^−1^[*z*/(1 + *z*)])],

where Z has an exponential distribution with the rate parameter *β*_1_, and

(17) $$\begin{array}{c} Ei\left(1,\beta_{1}\right)=\int_{\beta_{1}}^{\infty }\;\ln\left(z/\beta_{1}\right)e^{-z}dz. \end{array}$$

**Proof**

Let *f* be a pdf satisfying the requirements 1 − 3. The Kullback–Leibler divergence between *f* and *f*_*OWG*_ is given by

(18) $$\begin{array}{c} 0\leq D\left(f,f_{OWG}\right)=\int_{-\infty }^{\infty }f\left(x\right)\ln\frac{f\left(x\right)}{f_{OWG}\left(x\right)}dx\\ =\int_{-\infty }^{\infty }f\left(x\right)\ln\;f\left(x\right)dx-\int_{-\infty }^{\infty }f\left(x\right)\ln\;f_{OWG}\left(x\right)dx\\ =-S\left(f\right)-\int_{-\infty }^{\infty }f\left(x\right)\ln\;f_{OWG}\left(x\right)dx. \end{array}$$

For more details regarding Kullback–Leibler divergence between two arbitrary distributions, the reader is referred to Zografos and Balakrishnan [29] and references contained in it. Using the definition of the *f*_*OWG*_ as given in (4) and based on the constraints (1)–(3) yields

(19) $$\begin{array}{c} \int_{-\infty }^{\infty }f\left(x\right)\ln\;f_{OWG}\left(x\right)dx=\ln\;\beta_{1}+E_{f}\left[\ln\;g\left(x;\zeta \right)\right]-2E_{f}\left[\ln\bar{G}\left(x;\zeta \right)\right]-\beta_{1}E_{f}\left[\frac{G\left(x;\zeta \right)}{\bar{G}\left(x;\zeta \right)}\right]\\ =\ln\;\beta_{1}+E_{Z}\left[\ln\;g\left(G^{-1}\left[z/\left(1+z\right)\right]\right)\right]+2e^{\beta_{1}}Ei\left(1,\beta_{1}\right)-1\\ =-S\left(f_{OWG}\right). \end{array}$$

Substituting (19) into (18) yields

(20) $$\begin{array}{c} 0\leq D\left(f,f_{OWG}\right)=-S\left(f\right)+S\left(f_{OWG}\right), \end{array}$$

with equality if and only if *D*(*f*, *f*_*OWG*_) = 0, that is, if *f* = *f*_*OWG*_, which was to be proved.

As demonstrated by Lemma 3, the Shannon entropy of the OWG family in (4) is divided into two components. The first component is tied to the parameter *β*_1_ of the Weibull distribution, whereas the second part is entirely related to the arbitrary distribution *G*(*x*; *ζ*). Moreover, all members of the family in (4) share the first component and they are distinguished from each other solely by *E*_*Z*_[ln *g*(*G*^−1^[*z*/(1 + *z*)])]. Hence, the term *E*_*Z*_[ln *g*(*G*^−1^[*z*/(1 + *z*)])] can be used to distinguish between the members of the family in (4). It is possible, in some cases, to obtain an analytic form for the Shannon entropy of the family in (4), as shown in the following examples.

**Example 1 .**

Consider the odd Weibull uniform (OWU). The cdf and pdf of the uniform distribution is *G*(*x*; *η*) = *x*/*η* and *g*(*x*; *η*) = 1/*η*, respectively, where 0 < *x* < *η*. As a result *E*_*Z*_[ln *g*(*G*^−1^[*z*/(1 + *z*)])] = −ln *η*. Thus, using Lemma 3, The Shannon entropy of the OWU is given by

(21) $$\begin{array}{c} S\left(f_{OWU}\right)=1-\ln\;\beta_{1}-2e^{\beta_{1}}Ei\left(1,\beta_{1}\right)+\ln\;\eta . \end{array}$$

**Example 2 .**

As a second example, let us consider the odd Weibull exponential (OWE). The cdf and pdf of the exponential distribution is *G*(*x*; *λ*) = 1 − *e*^−*λx*^, and *g*(*x*; *λ*) = *λe*^−*λx*^, respectively, for 0 < *x* < *∞* and *λ* > 0. Consequently, *E*_*z*_[ln *g*(*G*^−1^[*z*/(1 + *z*)])] = ln *λ* − *e*^*β*_1_^*Ei*(1, *β*_1_). Thus using Lemma 3, the Shannon entropy of the OWE is given by

(22) $$\begin{array}{c} S\left(f_{OWE}\right)=1-\ln\;\beta_{1}-e^{\beta_{1}}Ei\left(1,\beta_{1}\right)-\ln\;\lambda . \end{array}$$

**Example 3 .**

Consider the odd Weibull logistic (OWL). This is obtained from (4) when the baseline distribution is the logistic distribution with cdf and pdf *G*(*x*) = 1/1 + *e*^−*λx*^ and *g*(*x*) = *λe*^−*λx*^/(1 + *e*^−*λx*^)^2^, for *x* ∈ *ℝ*, *λ* > 0, respectively. Then, *G*^−1^[*z*/(1 + *z*)] = ln(*z*)/*λ* and *g*(*G*^−1^(*z*/(1 + *z*)) = *λz*/(1 + *z*)^2^. Consequently,

(23) $$\begin{array}{c} E_{Z}\left[\ln\;g\left(G^{-1}\left[\frac{z}{\left(1+z\right)}\right]\right)\right]=\ln\;\lambda -\gamma -\ln\;\beta_{1}-2e^{\beta_{1}}Ei\left(1,\beta_{1}\right). \end{array}$$

Thus, the Shannon entropy of the OWL distribution is simply

(24) $$\begin{array}{c} S\left(f\right)=1+\gamma -\ln\;\lambda . \end{array}$$

Similarly, the Shannon entropy of other members of the family described in (4), including but not limited to, odd Weibull Pareto (OWP), odd Weibull half logistic (OWHL), odd Weibull power function (OWPF), and Odd Weibull Weibull (OWW) could be easily obtained via Lemma 3.

## 3. Discrimination Process

Consider a random sample *X*_1_, *X*_2_,…, *X*_*n*_ of size *n* from the OWG distribution with pdf in (4). The goal is to determine which model from the OWG family of distributions best fits a given dataset. To achieve this, we require a method for differentiating between various models within the OWG family. According to the maximum entropy principle, the most appropriate model to describe the data is the one with the distribution function *G* = *G*(*x*; *ζ*) that maximizes the corresponding Shannon entropy. In view of Lemma 3, to discriminate between two candidate models, with arbitrary cdfs *G*_1_ and *G*_2_ and respective pdfs *g*_1_ and *g*_2_, we use the following test statistic:

(25) $$\begin{array}{c} M_{1,2}=\frac{1}{n}\overset{n}{\underset{i=1}{\sum }}\left[\ln\frac{g_{2}\left(G_{2}^{-1}\left[z_{i}^{\left(2\right)}/\left(1+z_{i}^{\left(2\right)}\right)\right]\right)}{g_{1}\left(G_{1}^{-1}\left[z_{i}^{\left(1\right)}/\left(1+z_{i}^{\left(1\right)}\right)\right]\right)}\right], \end{array}$$

where

• *Z*_*i*_ = *G*_*j*_(*x*_*i*_)/1 − *G*_*j*_(*x*_*i*_), for *i* = 1, 2,…, *n*, and for arbitrary cdf *G*_*j*_ (where *j* = 1, 2).

### 3.1. Interpretation of Results

• If *ℳ*_1,2_ is positive, the test supports the model *OWG*_1_.
• If *ℳ*_1,2_ is negative, the test supports the model *OWG*_2_.

This procedure effectively discriminates between two models within the OWG family based on their respective cdfs *G*_1_ and *G*_2_.

### 3.2. Hypothesis Testing

To decide between the hypotheses:

• *H*_0_: parent distribution is *G*_2_
• *H*_1_: parent distribution is *G*_1_,

We use the statistic *ℳ*_1,2_. The null hypothesis *H*_0_ is rejected at a significance level *α* if

(26) $$\begin{array}{c} M_{1,2}\geq y_{\alpha }, \end{array}$$

where *y*_*α*_ represents the upper 100 × *α*% point of the distribution of *ℳ*_1,2_, under the null hypothesis *H*_0_.

## 4. Conclusions

In this article, the problem of discriminating between two members of the OWG family was considered. This discriminating process, using the method based on the maximum entropy principle, allows us to objectively decide which distribution from the OWG family best represents the data. It provides a robust statistical method for comparing two potential parent distributions by evaluating the entropy associated with each candidate.

## Data Availability

No data were used in the development of this study.
